# Anticipate study protocol: Baseline profile and care outcomes of patients attending Mater Misericordiae University Hospital with COVID-19 infection

**DOI:** 10.12688/hrbopenres.13091.2

**Published:** 2021-01-18

**Authors:** Gordana Avramovic, Tina McHugh, Stephen P Connolly, Walter Cullen, John S Lambert

**Affiliations:** 1 Mater Misericordiae University Hospital, Dublin, 44 Eccles St, Dublin 7, D07 AX57, Ireland; 2 University College Dublin, Catherine Mc Auley Centre, 21 Nelson St, Dublin 7, D07 KX5K, Ireland

**Keywords:** COVID-19, coronavirus, outcome, follow-up, care, hospital care, complications, virtual clinic

## Abstract

**Background:** While the COVID-19 pandemic is currently impacting on health and social care in Ireland, this impact is most marked in metropolitan Dublin. This is especially the case for the Mater Misericordiae University Hospital (MMUH) in Dublin’s North Inner, which is situated in an area where local socially deprived communities are at high risk of infection and of experiencing adverse outcomes.

**Aims: **To determine baseline characteristics and longer-term care outcomes of COVID-19 patients presenting to / attending the Infectious Diseases Department at MMUH, including the virtual clinic.

**Methods: **Retrospective study: we will retrospectively examine clinical records and extract anonymised data on patient demographics, baseline morbidity and outcomes.

Prospective study: we will prospectively examine healthcare outcomes among patients who consent to follow up at two time points (three months, and six months to 12 months after discharge/onset of disease). Two patient groups will be assessed for morbid complications: those hospitalised with COVID infection and those followed-up remotely with confirmed COVID infection.

**Deliverables:** The project will involve collaboration with Ireland’s Health Service Executive (HSE) Clinical Programmes and Ireland East Hospital Group to inform health service policies that will attenuate the adverse impacts of the COVID pandemic on population health. This research protocol will evaluate morbid complications of COVID depending on the severity of the disease.

## Introduction

Since March 2020, Ireland has experienced an outbreak of coronavirus disease 2019 (COVID-19), caused by severe acute respiratory syndrome coronavirus 2 (SARS-CoV-2). To date, little data have been reported on epidemiological and clinical characteristics of patients with COVID-19 in Ireland and internationally (especially in the European Union), therefore our knowledge in this regard remains limited. COVID-19 was declared a global pandemic on 11 ^th^ March 2020 ^1^. On May 28 ^th^, 2020 there were 5,488,825 confirmed COVID-19 cases (349,095 deaths) worldwide ^2^ and 24,841 confirmed cases (1639 deaths) in Ireland. In Ireland the median age of people infected with COVID-19 is 48, 57.2% of those infected are female, and infected persons most at risk of suffering severe illness and/or death are those over 65 years of age and those with underlying health conditions ^3^. Important information needs to be collected during and immediately following this emergency ^4^.

All of Ireland’s population are susceptible to COVID-19 infection ^5, 6^. Patients present with fever, cough, dyspnoea, fatigue, with some developing acute respiratory distress syndrome, multi-organ damage and secondary infection ^7, 8^. Symptoms of COVID-19 infection vary between individuals, and according to disease severity ^5, 7, 9^. Many are asymptomatic ^10^, some show mild to moderate symptoms ^5, 7^ although severe cases with acute respiratory distress syndrome, acute heart injury, and secondary bacterial infection have occurred ^5, 9^. COVID-19 infection is linked to a range of blood, cellular, and genetic abnormalities ^5, 7^. Those most at risk of severe illness as a result of infection include elderly males and/or people with underlying health conditions (e.g. diabetes, hypertension, heart disease, malnutrition) ^5, 10, 11^. Older age and elevated D-dimer levels are associated with adverse outcomes ^10^. Results of clinical trials will inform best treatment approaches but results of these are pending ^5^. A systematic literature review from China assessed the prevalence and severity of COVID-19 ^12^. Healthcare outcomes after two months were assessed in another systematic review in the Middle East. However, longer-term outcomes were not assessed past two months, and there is a need to continue to study long term morbid complications ^13^.

In Ireland, we are only just beginning to understand the epidemiology of COVID-19 at a whole population level. The burden of COVID-19 disease in the North Dublin community and initial manifestations and longer-term complications of COVID-19 infection need to be further delineated. The Mater Misericordiae University Hospital (MMUH) in Dublin is located in an area with high levels of social deprivation and a high incidence of COVID-19 infection. While some patients assessed have required acute care hospitalisation, others, with milder disease, have been placed on a 'virtual COVID community monitoring programme’ and managed in the outpatient setting. While COVID complications are likely to be more serious in those requiring hospitalisation, there is an evolving understanding that considerable morbidity can also occur in those with milder disease.

## Protocol

### Aims

We plan to establish and characterise the spectrum of disease and explore whether there are different clinical manifestations, and/or a greater risk of longer term complications in individuals who required hospitalization for COVID disease management; and individuals who tested positive and were supervised through the virtual COVID community monitoring programme. Results will be reported as a homogenous cohort.

### Setting

This longitudinal cohort study will take place at the Mater Misericordia University Hospital, in Dublin. To date, over 700 patients have been admitted to hospital or enrolled on the hospital’s virtual clinic / ambulatory home monitoring programme. Patients were admitted to hospital based on oxygen requirement at Emergency Room assessment. Our objective is to follow up these patients, at three months and between six and 12 months, to evaluate them for long term complications. At each visit we will review their quality of life and wellbeing, both medically and psychologically.

### Subject enrolment and consenting process

All patients who present or are referred during the COVID-19 outbreak will be eligible for the study. The requirement for consent was waived for the retrospective study by the ethics committee but is required for the prospective study. Data collection on patient deaths will be collected as part of the retrospective study.

At the outset, anonymised data will be retrospectively collected on all patients who were admitted from 1 ^st^ March 2020. No consent will be requested for the retrospective review. The data will be fully anonymized.

For the prospective study consent will be requested. When hospitalized patients are well enough and about to be discharged, they will be asked to participate in a prospective study including completing two interviewer-administered questionnaires and permit access to patient charts to review healthcare outcomes at three months and six to 12 months after discharge. For patients who have already been discharged, a member of the clinical COVID team will contact the patient by telephone to ask if they would be happy to be contacted to consider taking part in the follow-up study. If they accept, a consent form, patient information leaflet and a pre-stamped envelope for a return of correspondence will be sent out to them by post. Then a member of the research team will phone the patient to go through the consent form and information leaflet and answer any queries the patient may have regarding the study. If they decide to participate in the prospective study, participants will sign the consent form and it will be sent back to the study team via the pre-stamped envelope. A countersigned copy by authorised researchers will be posted back to them or given to them at their next attendance to the clinic. Virtual clinic patients are offered follow-up appointments after documentation of disease onset. When they present at the hospital for their follow-up appointment, a member of the research team will request their participation to the study. They will provide a patient information leaflet, give the patient time to read it and answer any questions. If the patient decides to participate to the study, they will sign an informed consent form ^14^.

### Sample size and power calculations

Based on current case reports from the Health Service Executive (HSE) Health Protection Surveillance Centre, we estimate that 500–1200 patients will present to the department during the study recruitment period. On 21 ^st^ June 2020, 798 patients had presented to the hospital (n=275) or virtual clinic (n=523) for COVID-19 assessment.

### Interventions to be measured - data collection and study instrument

A study Gantt chart is available in Table 1 outlining the study timeframe and interventions.

**Table 1.** Gantt chart: indicative timeline (by month from date of signature of Health Research Board agreement).

		**Month**	**1**	**2**	**3**	**4**	**5**	**6**	**7**	**8**	**5**	**6**	**7**	**8**	**9**	**10**	**11**	**12**	**13**	**14**	**15**	**16**	**17**	**18**
Coordination	1.1	*Project management*																						
	1.2	*Kick off meeting*																							
	1.3	*Steering committee*																						
	1.4	*Financial reports*																							
Dissemination	2.1	*Weekly reports UCD Medicine website - general public*																						
	2.2	*Reporting to HSE*																						
	2.3	*Publications x5*																						
Evaluation	3.1	*Review of project for data collection as pandemic evolves, project internal monitoring review*																						
	4.1	*Recruitment*																						
	4.3	*Retrospective review*																						
	4.2	*Prospective interviews and follow-up*																						
	4.2.1	*Questionnaire - Quality of Life Assessments- SF-12, PHQ2 measurement etc.*																						
	4.2.2	*Follow up of morbid complications via chart review. Data collected will include the following when applicable*																						
		*Pulmonary assessment: Pulmonary function testing (with diffusion capacity), six-minute walk test, Borg score*																						
		*Cardiology assessment: Echocardiography, ECG, NYHA score, BNP and troponin measurement (N.B. month six assessment only indicated if abnormality at month three)*																						
		*Neurological/neurocognitive assessment: Addenbrook cognitive assessment, history and examination*																						
		*Renal assessment*																						
		*Haematology assessment*																						
	4.5	*Data analysis*																						

UCD, University College Dublin; HSE, Health Service Executive; SF-12, 12-Item Short Form Health Survey; PHQ-2, Patient Health Questionnaire 2; ECG, electrocardiogram; NYHA, New York Heart Association; BNP, B-type natriuretic peptide.

1. Retrospective

Anonymised aggregated data will be collected on baseline demographics / health outcomes measures from clinical records, to include age, gender, type of health insurance, date presented, date admitted, date discharged, COVID test result, other illnesses at admission and laboratory and test results. A data collection instrument is included for reference and provides the details of data collected for the study. We will examine the factors that are associated with adverse initial outcomes – deaths while an in-patient, treatment in intensive care, longer hospital stays. Logistic regression analysis of clinical and demographic variables will be conducted in this regard.

2. Prospective

Study participants will be asked to complete two interviewer administered questionnaires (see *Extended data*) ^14^ at month three, and between month six and month 12 after discharge. The questionnaire will be administered in person or over the phone. They will report on their symptoms and quality of life through:

a) Study specific questions examining process of care (specifically referral / admission for assessment / treatment of an acute medical or psychological problem). Did they experience any health-related problems and if so, what were they?
b) General health status / quality of life will be assessed using the 12-Item Short Form Health Survey (SF-12), a multipurpose short-form generic measure of health status measuring eight domains: physical functioning, role limitations due to physical health problems, bodily pain, general health, vitality (energy/fatigue), social functioning, role limitations due to emotional problems and mental health (psychological distress and psychological well-being) ^15^.
c) Mental health symptoms, which are likely to be common at times of pandemic (especially depression / post-traumatic stress disorder) will be assessed using the Patient Health Questionnaire 2 (PHQ-2, a two-item screening questionnaire for depressive symptoms) ^16^, the four-item screening tool for Primary Care Post Traumatic Stress Disorder Screen ^17^ and the Depression and Anxiety Scales of PRIME-MD.
d) Some broad questions regarding alcohol use.
e) Borg questionnaire to measure breathlessness ^18^.

In addition consent will be requested to access medical records. Medical records will be analysed for morbid complications. Abnormal test results, as indicated by their COVID-19 care, will be recorded as relevant in the following categories: renal, respiratory, haematology, neurology and cardiology to provide a clinical picture of their health outcomes.

### Primary objectives

To establish the likelihood for complications in each patient group and assess it by type. To assess impact on patients’ overall quality of life and wellbeing, both medically and psychologically, at three and six to 12 months post infection by severity of disease. If clinically indicated we will review patients’ cardiovascular, neurological, renal, pulmonary and haematological status.

### Secondary objectives

Plans to follow-up future patients will be better defined. Quality of follow-up of patients will be improved.

### Data analysis and statistical plan

Frequency counts will be presented in respect of categorical and ordinal data and mean/median/range will be presented in respect of numerical data. We will examine how variables (such as age, gender, pre-existing morbidity, type of healthcare insurance and living in a deprived local area) are associated with outcome measures (admitted, discharged, readmitted within seven or 30 days, physical/mental health outcomes including morbid complications) using a modified logistic regression analysis. Inferential statistics will be used to examine relationships between variables.

With regards to data anonymisation, for the retrospective review of clinical records, all data will be anonymised at the time of data collection. Prospectively, all data will be coded and pseudonymised. Each participant will be given a numerical code, linked to their initials / date of birth and this will enable follow up. A list mapping these patient details (date of birth and initials) to numbers will be held by the research team. This list and all data will be stored as password protected files on a secure server at the UCD/MMUH Catherine McAuley Centre, 21 Nelson Street, Dublin 7. Study data will be retained for a period of five years after which it will be destroyed. Anonymised data will be made publicly available indefinitely.

### Research ethics approval and ethical considerations

Ethical approval was granted by the Institutional Review Board, Mater Misericordiae University Hospital, Dublin, Ireland on 8 ^th^ April 2020 (Ref # 1/378/2141).

With regards to consent, a member of the clinical team will provide potential participants with written information regarding the study, including the reason for the study, the nature of the questions to be answered, the interview methods chosen and how the data will be analysed and used. Given the potentially sensitive nature of the study, the decision for the initial approach to come from the health or social care professional, while not obligatory, is in accordance with advice from the Data Protection Agency, which recommends that a third party should request people to participate in research and should not “surprise” people consulting with health or other agencies. Those who are interested in participating will be asked to meet with a member of the research team. Upon speaking with a member of the research team, potential participants will be encouraged to express any remaining concerns or issues requiring clarification, before signing a consent form.

With regards to voluntary participation, it will be made explicit that non-participation in the study will not compromise the care they receive. No inducements to participate will be offered at recruitment.

If an individual is unable to read, two impartial witnesses must be present during the entire informed consent discussion. The individual must give consent orally and, if capable of doing so, complete, sign and date the form together with the person responsible for collecting the informed consent. If the patient is unable to sign or to mark a document so as to indicate that his or her consent is given clearly to the investigator or another member of the investigating team, the consent process must be conducted in the presence of two impartial witnesses present at the same time and recorded in writing.

The individual will be given one signed original informed consent form, the second original will be kept by the Principal Investigator. A copy will be placed in the patient’s medical record.

In designing this study, we have taken cognisance of best practice in conducting health research during times of major disasters ^4^.

### Plans for dissemination of the study outcome

The project will involve a collaboration with HSE Clinical Programmes to inform health service policies that will attenuate the adverse impacts of the COVID pandemic on population health. As such, outputs such as technical reports for stakeholders (at one, four, seven and 13 months) will be prioritised over the preparation of manuscripts for scientific journals, although such traditional outputs will also be disseminated.

Within one month of award, the University College Dublin (UCD) Medicine website will host information on the project and provide regular updates to the general public. A weekly email bulletin will be sent to stakeholder agencies to contain updated information on weekly/cumulative total number of cases, hospital admissions, discharges and re- admissions.

Five publications are expected from this work: general symptoms, pulmonary complications, cardiac complications, neurological complications and general quality of life.

The anonymized, FAIRified study data will be made available upon publication of research articles. Data will be made available via the Zenodo repository or a suitable repository will be chosen as advised by publishing journals. Study data for article published to date is available as a pre-print at: http://doi.org/10.5281/zenodo.4327641 ^19^.

### Study status

On the 29 ^th^ June 2020 the retrospective study had been completed for the first 100 patients and published ^19^. Amalgamation of retrospective results for a larger number of patients is in progress. The prospective study is recruiting patients and questionnaires are in progress.

## Data availability

### Underlying data

No underlying data are associated with this article.

### Extended data

Zenodo: Anticipate Study Baseline Profile and Care Outcomes of Patients attending Mater Misericordiae University Hospital with Covid-19 Infection. Zenodo. http://doi.org/10.5281/zenodo.4327641 ^14^

This project contains the following extended data:

- Questionnaire JL-COVID-19 version 3 dated 1 JULY 2020 clean.pdf
- Consent form COVID-19 version 3 dated 1 JULY 2020 clean.pdf
- Data collection sheet.doc

Data are available under the terms of the Creative Commons Attribution 4.0 International license (CC-BY 4.0).
