# Supplementary material for: Anticipate study protocol: Baseline profile and care outcomes of patients attending Mater Misericordiae University Hospital with COVID-19 infection
Source: HRB Open Res. 2021 Jan 18;3:52. Originally published 2020 Aug 7. [Version 2] doi: 10.12688/hrbopenres.13091.2 (PMC7888355; doi:10.12688/hrbopenres.13091.2)
Supplement: Peer review history

# Peer review: Reviewer response for version 2

**DOI:** 10.21956/hrbopenres.14340.r28670

**Published:** 2021-02-16

**Authors:** Desmond M. Murphy

**Affiliations:** 1 The Department of Respiratory Medicine, Cork University Hospital, Cork, Ireland

Improved for the changes. Would still like a comment or 2 on patients admitted for COVID, patients admitted with COVID due to inability to cope at home rather than the disease (and not because they were simply requiring oxygen) and whether pulmonary rehab was being offered to in-patients or those managed in the community or both. Clearly these factors will introduce issues with trying to interpret the relevance of any outcomes above and beyond the actual disease severity. While Borg and mMRC are potentially suitable, if not determining outcome mMRC as a ‘stand-alone’ classification may be more useful.

Is the study design appropriate for the research question?

Yes

Is the rationale for, and objectives of, the study clearly described?

Yes

Are sufficient details of the methods provided to allow replication by others?

Partly

Are the datasets clearly presented in a useable and accessible format?

Not applicable

Reviewer Expertise:

My research has examined airway remodelling in airways diseases including asthma and post-transplant obliterative bronchiolitis. My current research is mainly focused on asthma, in particular on how aspiration and differences in the microbiome influence airway inflammation and remodelling in severe asthma. I am a co-investigator on HRB-funded research examining the role of compliance and inhaler technique in asthma management.

I confirm that I have read this submission and believe that I have an appropriate level of expertise to confirm that it is of an acceptable scientific standard.

# Peer review: Reviewer response for version 2

**DOI:** 10.21956/hrbopenres.14340.r28669

**Published:** 2021-01-27

**Authors:** Jürgen Kurt Rockstroh

**Affiliations:** 1 Department of Medicine I, University Hospital Bonn, Bonn, Germany

The authors have successfully revised their article based on the comments made.

Is the study design appropriate for the research question?

Yes

Is the rationale for, and objectives of, the study clearly described?

Yes

Are sufficient details of the methods provided to allow replication by others?

Partly

Are the datasets clearly presented in a useable and accessible format?

Not applicable

Reviewer Expertise:

Infectious diseases.

I confirm that I have read this submission and believe that I have an appropriate level of expertise to confirm that it is of an acceptable scientific standard.

# Peer review: Reviewer response for version 1

**DOI:** 10.21956/hrbopenres.14192.r27959

**Published:** 2020-09-28

**Authors:** Desmond M. Murphy

**Affiliations:** 1 The Department of Respiratory Medicine, Cork University Hospital, Cork, Ireland

This study is timely and seeks to provide answers to important questions raised by the current COVID-19 pandemic. Within Ireland, COVID-19, was initially concentrated in Dublin and the surrounding areas although with clusters in other counties. However, more recently there appears to be increasing numbers nationally. Many Irish hospitals including The Mater have been affected. The data generated by this study will provide useful insight of both National and International relevance.

 My main concern is that the proposed methodology is vague and this aspect of the study would benefit from more detail.

 What about patient deaths - are these to be excluded and only survivors included? As written patients who die will not be captured as consent is essentially at discharge. This should be discussed.

 What severity scale of disease will be utilised to stratify patients and decide on admission or outpatient management?

 What scale will be utilised to dictate on-going patient management and categorise patients for data analysis?

 INEWS vs NCP/ITS Respiratory Scale or both (or other)?

 What severity of patients are being managed/proposed to be managed at home?

 A low INEWS score or all category A on NCP/ITS scale?

 What about patients managed virtually with subsequent admission?

 If pulmonary rehabilitation is being offered, is this to all patients or only those who have been admitted? How is it proposed to reflect this in final analysis?

 Why the Borg scale for breathlessness? Will mMRC be measured?

 The proposed outcomes are largely functional, e.g. breathlessness. This may be primarily pulmonary, cardiac or due to a myopathy. How do the authors propose to determine the possible aetiology of disability? Will patients be called for CT thorax, ECHO, 6 minute walk test etc? What point on the BORG or other scales will trigger potential referral and at what time point?

 The overall study is strong and relevant but in my opinion would benefit from addressing the issues highlighted.

Is the study design appropriate for the research question?

Yes

Is the rationale for, and objectives of, the study clearly described?

Yes

Are sufficient details of the methods provided to allow replication by others?

Partly

Are the datasets clearly presented in a useable and accessible format?

Not applicable

Reviewer Expertise:

My research has examined airway remodelling in airways diseases including asthma and post-transplant obliterative bronchiolitis. My current research is mainly focused on asthma, in particular on how aspiration and differences in the microbiome influence airway inflammation and remodelling in severe asthma. I am a co-investigator on HRB-funded research examining the role of compliance and inhaler technique in asthma management.

I confirm that I have read this submission and believe that I have an appropriate level of expertise to confirm that it is of an acceptable scientific standard, however I have significant reservations, as outlined above.

# Response

**Published:** 2020-12-07

**Authors:** John Lambert

**Affiliations:** Mater Misericordiae University Hospital, Dublin, Ireland

*This study is timely and seeks to provide answers to important questions raised by the current COVID-19 pandemic. Within Ireland, COVID-19, was initially concentrated in Dublin and the surrounding areas although with clusters in other counties. However, more recently there appears to be increasing numbers nationally. Many Irish hospitals including The Mater have been affected. The data generated by this study will provide useful insight of both National and International relevance.*

*My main concern is that the proposed methodology is vague and this aspect of the study would benefit from more detail.*

*What about patient deaths - are these to be excluded and only survivors included? As written patients who die will not be captured as consent is essentially at discharge. This should be discussed.*

*What severity scale of disease will be utilised to stratify patients and decide on admission or outpatient management?*

 In response to reviewer 2, we have made the following changes:

 More detail has been added to the methodology. Data on deaths during hospital will be documented.  For the retrospective study consent is not necessary as per current ethical approvals. Consent is necessary for the prospective study including study questionnaires.

*What scale will be utilised to dictate on-going patient management and categorise patients for data analysis?*

 Replaced ‘severe COVID 19 with the terms ‘patients who required hospitalization’. We will deal with the issue of patients who were attending the clinic for virtual monitoring who subsequently required hospital admission as follows: The cohort of all patients will be reported as a homogenous cohort.

 We will examine the factors that are associated with adverse initial outcomes – deaths while an in-patient, treatment in intensive care, longer hospital stays. Logistic regression analysis of clinical and demographic variables will be conducted in this regard.

*INEWS vs NCP/ITS Respiratory Scale or both (or other)? A low INEWS score or all category A on NCP/ITS scale? If pulmonary rehabilitation is being offered, is this to all patients or only those who have been admitted? How is it proposed to reflect this in final analysis?*

 Pulmonary rehabilitation is being offered as part of the clinical care patients receive and thus, if clinically indicated as determined by the treating medical team. We will collect available data on Oxygen supplementation. There are no interventional arms in this study. The aim as I see it is to characterise the spectrum of disease. Neither the primary nor secondary end points are looking at the effects of rehabilitation.

*What severity of patients are being managed/proposed to be managed at home? What about patients managed virtually with subsequent admission?*

 Patients with no oxygen requirements were managed from home. Assessed as stable in the Emergency Room. The patient cohort is dealt with as a homogenous cohort.

*Why the Borg scale for breathlessness? Will mMRC be measured?*

 Both are validated and acceptable for a study such as this. We have opted for the Borg scale.

*The proposed outcomes are largely functional, e.g. breathlessness. This may be primarily pulmonary, cardiac or due to a myopathy. How do the authors propose to determine the possible aetiology of disability? Will patients be called for CT thorax, ECHO, 6 minute walk test etc? What point on the BORG or other scales will trigger potential referral and at what time point?*

 This study is non interventional, the aim is to collect data. If patients in the 2 groups (Admitted Vs non Admitted) have wildly different symptomatology and QoL on follow up then further work to determine the aetiology would seem feasible.

# Peer review: Reviewer response for version 1

**DOI:** 10.21956/hrbopenres.14192.r27788

**Published:** 2020-08-17

**Authors:** Jürgen Kurt Rockstroh

**Affiliations:** 1 Department of Medicine I, University Hospital Bonn, Bonn, Germany

The researchers plan to establish and characterize the spectrum of COVID-19 disease and explore whether there are different clinical manifestations, and/or a greater risk of longer term complications between two groups: 1) individuals admitted to hospital for more severe COVID disease management; and 2) individuals who tested positive and were supervised through the virtual COVID community monitoring program.

 It may be more pragmatic to simply study all hospitalized patients (regardless whether defined as more severe or not) versus all individuals who were monitored through the community program. However, if the definition “severe COVID-19” is used it should be defined more clearly. Also there is a need to explain how the researchers will deal with patients who started off with a virtual community monitoring but with more progressive disease were then hospitalized. Obviously they still should stay in the monitoring group rather than then adding them to the hospitalized group.

 Clearly this study is very timely as the COVID-19 pandemic is unfolding and many clinical and epidemiological questions are still unanswered.

 Nevertheless, the overall proposal is very general and would really need some more detailed description of what kind of clinical and lab data will be collected or is available. It is stated that “….other illnesses at admission and laboratory and test results…." will be captured but what diseases and what lab values are they talking about? This really would need to be defined upfront. How will the researchers deal with certain diseases or co-infections which may warrant additional data. For example in case of HIV co-infection will there be an attempt to gather additional data on CD4 count or viral load? What about co-medications which are supposingly affecting risk or outcome of COVID-19 disease; will they be captured as well?

 A strength of the proposal is the use of standardized questionnaires to capture the impact of a COVID-19 infection on general health and mental well-being.

Is the study design appropriate for the research question?

Yes

Is the rationale for, and objectives of, the study clearly described?

Yes

Are sufficient details of the methods provided to allow replication by others?

Partly

Are the datasets clearly presented in a useable and accessible format?

Not applicable

Reviewer Expertise:

Infectious diseases.

I confirm that I have read this submission and believe that I have an appropriate level of expertise to confirm that it is of an acceptable scientific standard, however I have significant reservations, as outlined above.

# Response

**Published:** 2020-12-07

**Authors:** John Lambert

**Affiliations:** Mater Misericordiae University Hospital, Dublin, Ireland

In response to reviewer 1, we have made the following changes:

 •             Replaced ‘severe COVID 19’ with (as appropriate) the terms ‘patients who required hospitalization’ or ‘patients requiring admission to intensive care’ or ‘patients requiring longer hospital stay’

 •             We will deal with the issue of patients who were attending the clinic for virtual monitoring who subsequently required hospital admission as follows: The cohort of all patients will be reported as a homogenous cohort.

 •             Clinical and lab data to be collected is defined in the data collection instrument now included, and now summarized in the text of the paper. We are not collecting lab data outside of that defined in the paper. Data on medicines prescribed is being collected.
